# A Late and Complex Presentation of Hereditary Haemochromatosis

**DOI:** 10.7759/cureus.32025

**Published:** 2022-11-29

**Authors:** Roshini Kurian, Preethu Anand, George Ghaly

**Affiliations:** 1 Internal Medicine, North Cumbria Integrated Care, NHS Foundation Trust - Cumberland Infirmary, Carlisle, GBR; 2 Internal Medicine, University Hospitals of Leicester, NHS Trust - Leicester Royal Infirmary, Leicester, GBR; 3 Geriatrics, University Hospitals of Leicester, NHS Trust - Leicester Royal Infirmary, Leicester, GBR

**Keywords:** hfe gene mutation, primary iron overload, myelodysplastic (mds)/myeloproliferative neoplasm (mpn) disease spectrum, liver abscess, congestive cardiac faliure

## Abstract

We report a case of a 78-year-old male with a complex presentation that first diverted our attention from the underlying hereditary haemochromatosis (HH). A fit patient who initially came with leg pain and eventually died within 3 months of presenting with several syndromes relatable to HH that uncommonly manifest together. His initial presentation was pyomyositis in the thigh muscles followed by a diagnosis of myelodysplasia - refractory anaemia with excess blasts (RAEB), congestive cardiac failure and liver abscesses. End-stage heart failure and recurrent infections were the main causes of the patient’s death prior to trials of specific treatment for HH.

Recurrent atypical infections and myelodysplastic syndrome (MDS) should raise alarms for iron overload. In HH there can be a rapid progression of the disease process resulting in nearly irreversible organopathy, thus impeding treatment trials. Early detection and reduction of iron overload may reduce morbidity and mortality.

## Introduction

Hereditary haemochromatosis (HH) is one of the most common inherited diseases with a prevalence of 1 in 300 to 500 individuals. HH types 2, 3, and 4 are seen worldwide however, type 1 which is linked with HFE gene mutations is mostly seen in people of Northern European descent. Men are affected twice as much as women. In men, the disease becomes evident in the fifth decade of life while in women it becomes apparent in the sixth decade of life. 

Primary haemochromatosis is a genetic disorder of iron metabolism resulting in tissue iron overload that usually ends in multiorgan involvement. Iron overload can also be the result of various secondary causes, the most common of which is repeated blood transfusions.

The common presentations of iron overload are those of hepatic dysfunction, cardiac involvement, and diabetes. While rare presentations with multisystem involvement should raise alarms to investigate and establish the diagnosis of haemochromatosis, this can be challenging given the nonspecific and subtle features initially. Timely recognition is vital in initiating interventions early on.

## Case presentation

**Figure 1:**
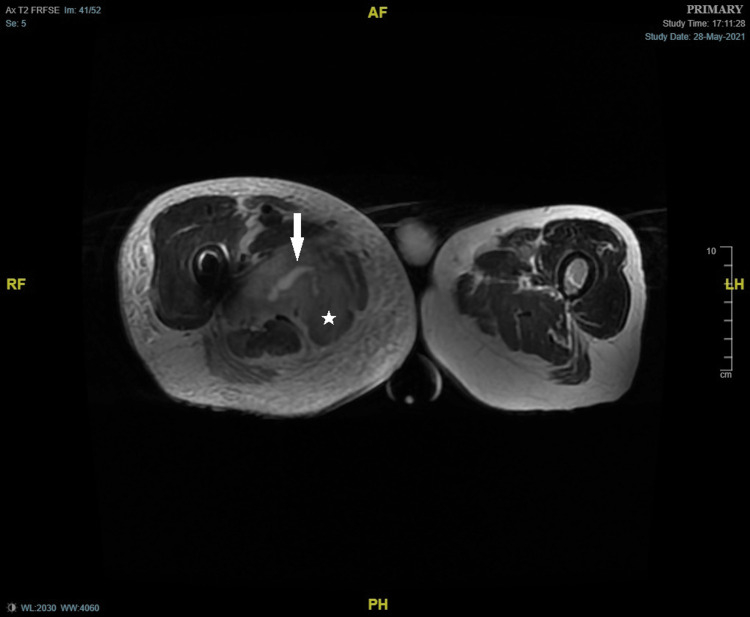
MRI thigh - T2-weighted image Arrow:  Muscle collection; Star: Tissue oedema

**Figure 2:**
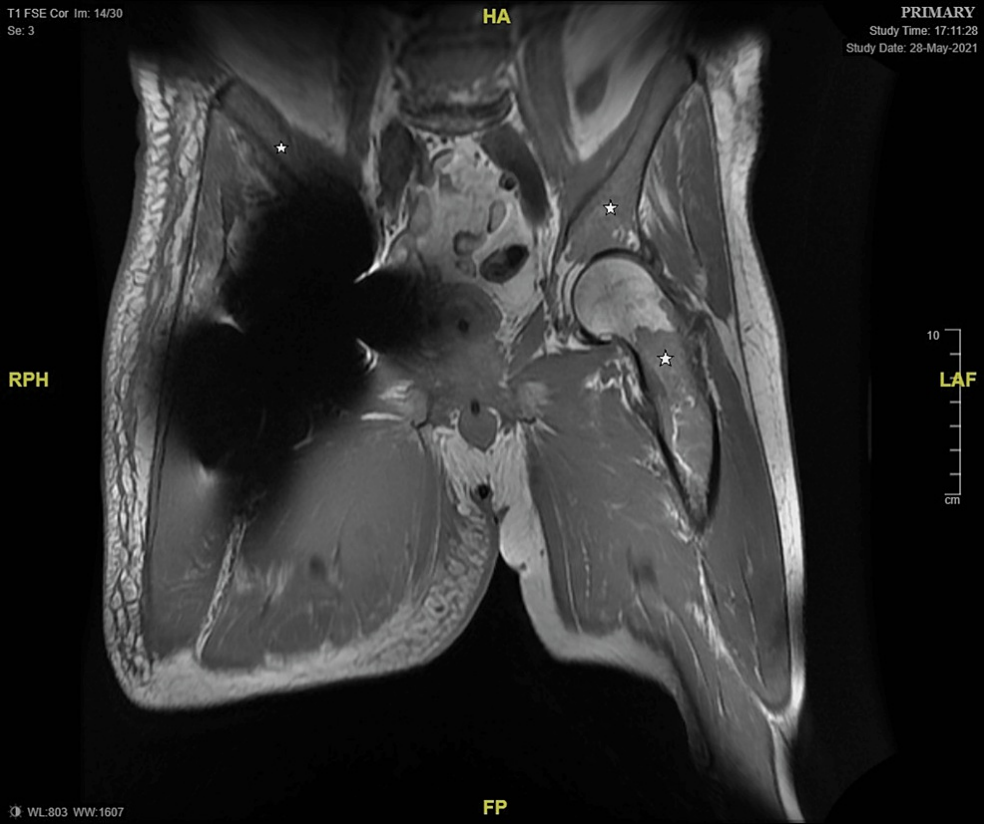
MRI hip - T1-weighted image Star: Marrow hyperplasia of pelvis and femur

A 78-year-old male, with a past medical history of atrial fibrillation and right total hip replacement, first presented to the hospital afebrile, tachypneic with swelling and redness of the right infra-gluteal area. His initial laboratory work-up showed abnormal biomarkers - total white cell count (WCC) 20.2 x 10^9^/L (4 - 11), haemoglobin (Hb) 104 g/L (130 - 180), Platelet count (PC) 412 x 10^9^/L (140 - 400), C-reactive protein (CRP) 350 mg/L (0 - 10). Both CT and MRI hip suggested infective myositis or pyomyositis with intramuscular collections within the right adductor magnus, the largest pocket measuring 14 mm x 50 mm, and showed red marrow hyperplasia of the imaged spine, pelvis, and proximal femur (Figures 1-2).

He was admitted to an orthopaedic ward and treated with intravenous antibiotics for the myositis. As he responded to antibiotics, the plan of ultrasound-guided aspiration of the muscle collection was withheld. In light of MRI findings of red marrow hyperplasia and peripheral smear findings of dyserythropoesis suggested by the presence of stomatocytes, occasional nucleated red blood cells, polychromasia and anisocytosis; bone marrow aspiration and biopsy were performed which reported markedly hyperplastic particles with cellular trails, increased and dysplastic megakaryocytes, markedly reduced erythropoiesis, increased and dysplastic granulopoiesis with left shift and hypogranulation, blasts of 14% which were small with very high nucleus-to-cytoplasmic ratio and visible nucleoli, reduced and mature appearing lymphocyte with normal iron stores (grade 3/6) on aspirate. This was suggestive of refractory anaemia with excess blasts (RAEB) according to World Health Organisation myelodysplastic syndrome (MDS) classification. Cytogenetics of the bone marrow biopsy sample showed karyotype - 46, XY(20). Of note, the patient did not have previous haematological records or records of blood transfusions in the past. Further, in the admission, the patient’s Hb dropped to 86 g/L and a haematinic profile at that time showed a transferrin saturation of 82% (20 - 55) with a ferritin level at 1433 mcg/L (23 - 540). An outpatient haematology follow-up was planned, however, this did not happen as the patient presented rapidly again to the hospital.

The patient presented to ED, a few days later, with signs of chest infection, nausea and dehydration. He improved with antibiotics and was discharged within a week.

**Figure 3:**
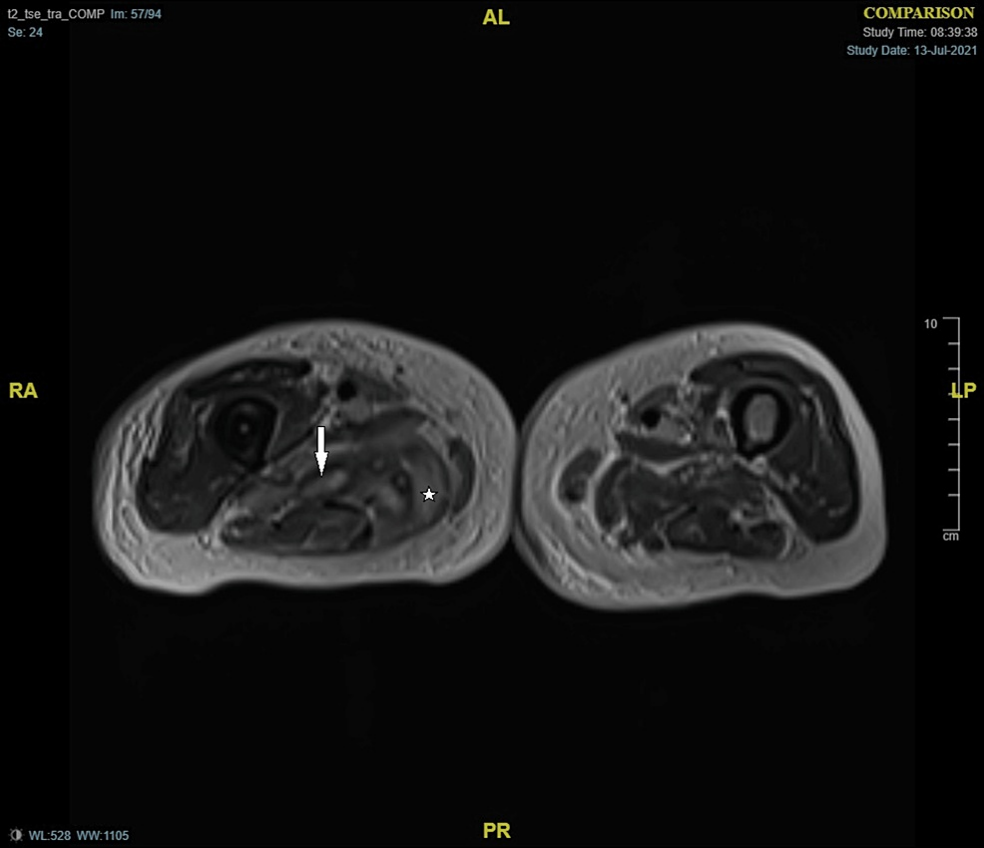
MRI thigh - T2-weighted image Arrow: Muscle collection; Star: Tissue oedema

**Figure 4:**
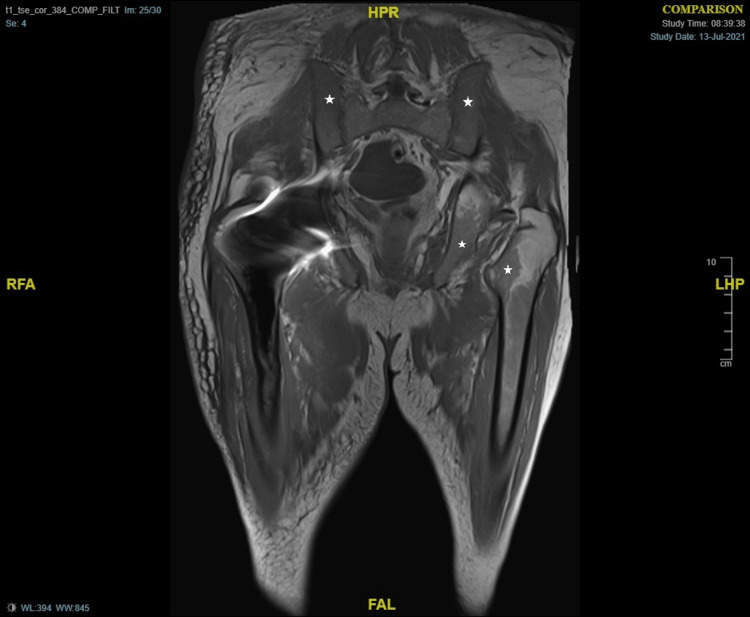
MRI hip - T1-weighted image Star: Marrow hyperplasia of pelvis and femur

He was re-admitted again with diarrhoea and features suggestive of fluid overload. On clinical examination, he was febrile and had gross oedema, tachypnoea, tachycardia with irregularly irregular pulse, decreased air entry in lung bases. Blood picture showed Hb 86 g/L, PC 645 x 10^9^/L, WCC 12.4 x 10^9^/L, CRP 124 mg/L, N-terminal pro B-type natriuretic peptide (NT pro-BNP) 4798 ng/L (20-200). A diagnosis of congestive cardiac failure, and anaemia on the background of myelodysplastic syndrome was made with the suspicion of ongoing pyomyositis, and he was treated with intravenous diuretics and antibiotics. He continued to remain febrile while his blood cultures showed no growth. A transthoracic echocardiogram showed dilated heart chambers except for the left ventricle and moderate biventricular failure. Repeat MRI pelvis and hip showed marginal regression of the previous muscle collection yet with more generalized muscle and subcutaneous oedema (Figures 3-4).

An autoimmune screen for polymyositis, vasculitis and other relevant conditions came back negative. Myeloma studies showed a rise in immunoglobulins with no monoclonal gammopathy. The patient had limited improvement in his decompensated heart failure and his haemoglobin dropped gradually to 79 g/L.

**Figure 5:**
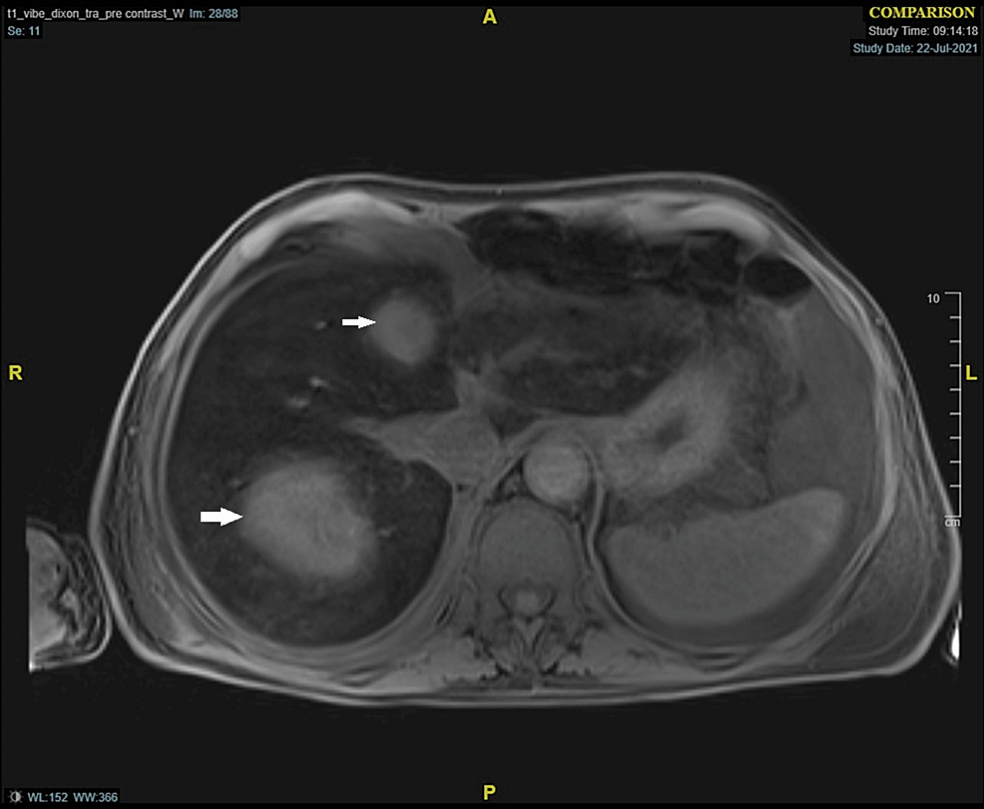
MRI liver Arrow: Liver abscess

As the patient was still febrile despite being on broad-spectrum antibiotics, further investigations were performed. Whole body CT scan showed multiple rim-enhancing lesions in the liver, likely liver abscesses, which was subsequently confirmed by MRI (Figure 5). The MRI also suggested background liver features of iron overload. Following this liver biopsy was performed. Histology reported three brown and pale cores. Consisting predominantly of fibrinous and necrotic material with admixed inflammatory cells, no malignant cells were seen. Iron staining showed significant iron deposition within hepatocytes. Trichrome highlighted bridging fibrosis that was difficult to grade as the available sample was scanty. Liver abscess aspirate could not be cultured due to inadequate sampling.

Iron panel showed transferrin saturation of 27% and ferritin 5000 mcg/L and subsequently 15300 mcg/L, with satisfactory B12 and folate levels. Liver functions showed rising trends of alkaline phosphatase and bilirubin while alanine transaminase was at baseline. In view of the MRI findings of increased iron deposits in hepatocytes and the iron panel, HFE gene studies were sent which reported two copies of the HFE variant p.(Cys282Tyr) (C282Y).

With ongoing management of refractory heart failure, treatment of infections with prolonged antibiotic courses and aspiration of liver abscesses there was no room to stabilize the patient and initiate treatment for the underlying iron overload. The patient was not a candidate for venesection given anaemia and MDS. The alternative in such a case would have been iron chelation, but with the limitations that injectable iron chelators (desferoxamine) favour the growth of siderophoric organisms and oral chelators (deferiprone, deferasirox) may cause agranulocytosis and neutropenia.

Due to multi-organ dysfunction and clinical deterioration despite all efforts, the focus was supportive care. The patient passed away in comfort.

## Discussion

Hereditary haemochromatosis is an autosomal recessive disorder characterised by increased intestinal iron absorption that results in iron overload. It is now clear that this happens through hepcidin deficiency or even resistance. The two common HFE gene mutations responsible are located on chromosome 6 and are now named C282Y and H63D. [1]

6% of the normal Caucasian population carries one copy of the C282Y gene [2]. Over 90% of UK HH patients have two copies of the C282Y variant and less than 5% are heterozygotes carrying one copy of the above-mentioned mutations [3]. HFE gene analysis of our patient showed homozygous p.(Cys282Tyr) mutations.

Research now describes in more detail the toxicity of iron on the intracellular structures by creating a highly oxidative stress process ending in cytotoxicity and cell death described now in the heart and liver by the term ferroptosis. More literature is now showing this stage is irreversible highlighting the importance of early intervention.

This patient presented initially with atypical, localized myositis like the picture on MRI showing a pus collection. No specific organism was isolated. Later on, he developed necrotic liver abscesses and initially on both occasions the patient seemed to respond to broad-spectrum antibiotics, with a rise in inflammatory markers intermittently. Both iron-enriched and oedematous tissues from the congestive state are favourable media for certain pathogens. *Yersinia *is one such iron-dependent bacterium that relies entirely on exogenous iron for growth. There have been case reports of six patients presenting with abdominal pain and deranged liver function, in whom imaging revealed liver abscesses colonised by *Yersinia enterocolitica *on the background of iron overload and led to the diagnosis of haemochromatosis. [4-7] However, in this case, we have been unable to culture the organism from the liver aspirate.

Haemochromatosis is associated with an increased risk of haematological neoplasia, including MDS [8]. This has been well studied in cases of secondary haemochromatosis due to repeated blood transfusions, as in patients with known inherited anaemia, but there is now a growing interest in studying the links between HFE gene mutations and haematological neoplasia, especially MDS. The onset of MDS in haemochromatosis usually occurs between 60 and 70 years of age, while cases with advanced age are very rare [9]. Our patient did not have a past history of chronic cytopenias or multiple blood transfusions. The preliminary investigations in our patient revealed dyserythropoiesis and a bone marrow analysis confirmed the diagnosis. A diagnosis of MDS should warrant investigating for HH and vice versa due to its implication on management plans and prognosis.

Elevated fasting transferrin saturation (TSAT) is the phenotypic hallmark of the disorder. Several other conditions may also affect TSAT by modulating the rate of transferrin synthesis. The presence of infection, inflammation, and chronic disease downregulate its synthesis. TSAT levels are also reduced in malignant conditions and malnutrition [10]. This could likely explain the unexpected drop in transferrin saturation of our patient further in the course of the disease process. Hence, to prevent misinterpretation and erroneous conclusions, great attention should be paid to the inherent limitations of this test.

There have been case reports regarding cardiac complications of primary and secondary haemochromatosis [11-13]. Cardiac iron deposition starts from the epicardium inwards with clinical implications depending on the area involved. Supraventricular and AV (atrioventricular) block arrhythmias are common arrhythmia-related complications and can happen prior to significant structural changes, but the biventricular involvement and the resulting dysfunction are the leading cause of morbidity and mortality in HH. Restrictive pattern cardiomyopathy usually precedes a stage of dilated overt dysfunction by which time the chance of reversibility is extremely limited, yet early reduction of iron overload carries the only chance to improve the outcome, and cardiac transplantation should be an early consideration in appropriate candidates [14].

The mainstay of treatment in haemochromatosis is reducing iron overload. Iron-depleting therapy in patients with iron overload has shown a significant improvement in survival and quality of life up to comparable numbers to the general population in the 21st century. There is also a recognised but much more limited improvement in survival in patients with established organopathy predominantly cirrhosis and cardiomyopathy [15,16]. Iron chelation with desferrioxamine and deferiprone is an alternative in patients with anaemia or where phlebotomy is contraindicated. However, desferrioxamine can cause immunological compromise and thereby increase susceptibility to Yersiniosis by making the host’s iron more bioavailable [17]. While deferiprone could be used in this instance as it has a comparable efficacy as desferrioxamine at similar doses yet the most common side effect with it is agranulocytosis. Studies have shown that where monotherapy is not tolerable or effective, combination therapy is proven to be more effective in the management of severe cardiac siderosis [18].

Hereditary haemochromatosis was long thought to be a clinically and genetically distinct condition characterised by classic presentation with hyperglycemia, bronze skin pigmentation, and cirrhosis, while in fact, it is protean in its manifestation. Our case serves as an example that presentations might be unusual, complicated and delayed. After a thorough review of the scientific literature in PubMed and Open Athens Research Hub from the years 1978 to 2022, we came across only one other case report of HH that presented with multisystem involvement - left ventricular failure, hepatomegaly, diabetes, hypogonadism and arthralgia [19]. This highlights how distinctive this paper is and urges physicians to keep an open mind while reviewing complex presentations of a known condition.

## Conclusions

Hemochromatosis should be considered as a differential in complex presentations with multi-organ involvement. Initiating treatment at the early stages of hemochromatosis prior to end-organ damage may help reduce morbidity and mortality. The challenge always remains with picking up the earliest of signs.
